# *Piriformospora indica* enhances growth and salt tolerance in a short rotation woody crop, *Paulownia elongata*, under NaCl stress

**DOI:** 10.3389/fpls.2025.1566470

**Published:** 2025-06-23

**Authors:** Deyu Mu, Meng Zhang, Yu Liang, Chen Ding, Qinglei Chen, Xiaoli Fan, Xiang Meng, Xinpeng Zhang, Shengyu Gao, Datong Zhai, Yubo Gao, Yawei Wu

**Affiliations:** ^1^ Lab of Ornamental Plants, Shandong Jianzhu University, Jinan, China; ^2^ Wetland Research Institute, Shandong Academy of Forestry Sciences, Jinan, Shandong, China; ^3^ College of Forestry, Wildlife and Environment, Auburn University, Auburn, AL, United States; ^4^ Institute of Botany, Jinan Academy of Landscape and Forestry Sciences, Jinan, Shandong, China

**Keywords:** *Paulownia elongata*, *Piriformospora indica*, antioxidant oxidase, salt tolerance, osmotic adjustment substance

## Abstract

Salinization is a major environmental challenge that jeopardizes productivity and resilience of plants such as the short rotation woody crops (SRWC) and bioenergy crops. Leveraging beneficial microbes will enhance plant resistance to salinity with physiological adjustments. Here we investigated the efficacy of plant growth promoting fungus (*Piriformospora indica*) on optimizing growth and salt tolerance of SRWCs and bioenergy tree crops, using *Paulownia elongata* as an example. Following culture in sterile soil, the chlamydospore of *P. indica* were found in paulownia plants roots. We treated both inoculated and uninoculated plants with four salt concentrations (0.00%, 0.30%,0.50%, 0.70%) by soaking them in varying concentrations of NaCl solution every 7 days. After 30 days of treatment, we investigated various physiological parameters, i.e., biomass, infection rate, growth rate, photosynthetic parameters, antioxidant enzyme activity, and soluble sugar of paulownia plants. Our two-way ANOVA demonstrated that the interaction between salinity stress and *P. indica* inoculation significantly enhanced plant height growth rate, leaf net photosynthetic rate, superoxide dismutase (SOD) activity, and soluble protein content in *Paulownia* seedlings. Inoculated plants exhibited improved salt tolerance due to the mitigating effect of symbiosis across a salinity gradient. Mortality in the *P. indica*-treated group was reduced by approximately 5.55%, 22.22%, and 27.77% under 0.30%, 0.50%, and 0.70% NaCl treatments. Our study is the first application of *P*. *indica* to enhance salinity tolerance in *Paulownia*, a short-rotation woody crop. Inoculating such endophyte significantly improves the resilience and productivity of *Paulownia* plantations in saline environments, for a sustainable afforestation effort.

## 1 Introduction

Soil salinization is a critical global ecological challenging, affecting over one billion ha^2^ of terrestrial land and leading to land degradation, declining agricultural productivity ecosystem functions, as well as long-term threats to food security and socio-economic stability (Negacz et al., 2022). Salt accumulation in soil increases rhizosphere osmotic pressure, disrupts the plant’s water retention, reduces water uptake capacity, and limits nutrient availability. Salinity stress inhibits plant height growth, causes tissue damage in leaves, and alters biomass accumulation and distribution, compromising overall plant health and productivity (Yadav et al., 2019; He et al., 2022; Ma et al., 2023).

Salinity stress can be divided into two parts: in the short term, elevated NaCl concentrations in the root zone impair water uptake by plants, resulting in osmotic stress and growth inhibition (Acosta-Motos et al., 2017). During the increase of salt stress time, excessive salt ions entering the transpiration flow of plants through roots are transported to branches and leaves, and inhibit photosynthesis occurs and ion toxicity, subsequently inhibiting plant growth (Parihar et al., 2015). In addition to osmotic stress and ion toxicity, salt stress also induces oxidative stress, resulting in membrane lipid peroxidation (Acosta-Motos et al., 2017). Plants eliminate oxygen free radicals through the antioxidant enzyme system, preventing oxidative damage to cells for survival in adverse environments. The antioxidant enzymes will be adjusted under the salinity mainly including superoxide dismutase (SOD), peroxidase (POD) and catalase (CAT) (Muchate et al., 2016). The activities of SOD, POD and CAT are higher in the leaves of alfalfa (*Medicago sativa*) treated with mixed saline-alkali at seedling stage with the increase of mixed saline-alkali concentration and were higher than those of the control (Al-Farsi et al., 2020). Under certain levels of salinity conditions, the plant itself can eliminate the influence of adversity environment by increasing the activities of SOD, POD and CAT to improve the stress tolerance. However, with a gradual increase concentration of salinity, the activities of SOD, CAT and POD decreased, and the oxygen free radicals cannot be eliminated, resulting in the damage of cell membrane and finally the plant death (Zhang et al., 2013). Plants also alleviate the physiological damages due to salinity stress by regulating the content of endogenous osmotic substances (Wang et al., 2022; Feng et al., 2023). Soluble sugar content as an index reflects the tolerance of plants to salt stress (Saied et al., 2005). Previous studies showed that the level of reducing sugar while sucrose in mulberry (*Morus alba*) significantly increases at a 150 mM salt concentration (Liu et al., 2019).

Enhancing woody plants’ resilience is key for a sustainable development of bioeconomy (Seth, 2003) ecosystem conservation. Mitigating saline wastewater using short rotation woody crops (SRWC) has been tested and applied for phytoremediation (Dimitriou et al., 2006; Zalesny et al., 2007; Mirck and Zalesny, 2015; Pradana et al., 2023). Fast-growing *Paulownia Clon* in Vitro 112 is a new bio-energy crop in Europe for fuel production (Świechowski et al., 2019). Improving our knowledge of salt tolerance of short rotation woody crops (SRWC) is critical relative to most other economically important species. Such knowledge gap will slow down the broad deployment of SRWC especially at challenging sites.

In the early 19th century, paulownia (*Paulownia elongata*) was introduced as an ornamental tree species by the United States and some European countries from China (Snow, 2015). Later, some researchers found that its commercial value could be widely used in the production of various wood commodities such as musical instruments, sculptures, and furniture (Özelçam et al., 2021). Paulownia improves environmental quality through significant absorption of carbon dioxide, sulfur dioxide, and airborne particulate matter (Osmanović et al., 2017). Paulownia is also an excellent garden greening tree species with high economic, ecological, and social benefits because of its tall canopy, wide crown, diverse flower color and fragrant blooms. (Woźniak et al., 2022). found that Paulownia has immense potential in the transformation of abandoned land and the improvement of soil quality. It can increase soil enzyme activity, microbial biomass and microbial metabolic diversity, and is a bioenergy crop (Woźniak et al., 2022). Paulownia provides energy regeneration in short-term, which can be harvested and can regenerate every 3 to 5 years in about 25 years rotation (Abreu et al., 2022). Despite its economic promise, Paulownia’s cultivation is limited to non-saline soils, as its salt tolerance thresholds and physiological adaptations remain uncharacterized. The current research on paulownia emphasizes using paulownia wood (Jakubowski, 2022), breeding salt-tolerant varieties of paulownia (Ayala-Astorga and Alcaraz-Melendez, 2010), and improving the wood properties (Kaygin et al., 2009). The mechanisms underlying salt tolerance in Paulownia and strategies for its enhancement remain poorly understood. (Ivanova et al., 2019). investigated the effects of different salinity on the growth, leaf anatomical structure and gas exchange characteristics in two *Paulownia* hybrid lines (*Paulownia tomentosa 9 fortunei-TF* and *Paulownia elongata 9 elongata-T4*) by pot experiments and reported that compared with T4, the total dry biomass, leaf area, total leaf area/leaf number ratio and leaf K^+^/Na^+^ ratio of TF01 decreased more significantly under an elevated the soil salinity. Genetic research showed higher salt tolerance in polyploidization too (Deng et al., 2017; Cseri et al., 2020).

*Piriformospora indica* is an endophytic fungus obtained by Verma et al. in 1998 from the roots of shrubs in the Thar Desert, India (Varma et al., 1999). The endophytic fungus *Piriformospora indica* was selected for this study due to its proven efficacy in enhancing abiotic stress tolerance across diverse plant species; It can colonize the roots of diverse plant species, enhancing growth and improving stress tolerance in host plants under various abiotic stress conditions (Johnson et al., 2014), including drought (Boorboori and Zhang, 2022), salinity (Sabeem et al., 2022), waterlogging (Li et al., 2023), low temperature (Liang et al., 2022), heavy metal (Singhal et al., 2017) etc. The biomass of maize (*Zea mays*), tobacco (*Nicotiana tabacum*), soybean (*Glycine max*) significantly increased after colonization by *P. indica* compared to non-colonized control plants (Sharma, 2008). (Liang et al., 2023). demonstrated that the endophytic fungus *P. indica* enhances the waterlogging tolerance in peach (*Prunus persica*) seedlings by increasing the activity of key antioxidant enzymes (Liang et al., 2023) to enhance physiological resilience under anaerobic conditions. *P. indica* can also enhance the cold resistance in banana (*Musa nana*) by reducing malondialdehyde (MDA) content, promoting antioxidant enzyme activity via accumulating soluble sugar and increased proline content, up-regulating the expression of cold-responsive genes in banana leaves (Li et al., 2021); thus, these physiological adjustments collectively improve the plant’s cold adaptation. (Su et al., 2021). studied the effect of endophytic fungus *Piriformospora indica* on alleviating cadmium (Cd) stress in tobacco (*Nicotiana tabacum*) and found that *P. indica* enhanced the tolerance of tobacco to Cd by increasing Cd accumulation in tobacco roots, while reducing the Cd translocation and accumulation in leaves; thus the above-ground tissue has limited toxicity by root sequestration (Su et al., 2021). (Xu et al., 2017). showed that *Piriformospora indica* inoculation in maize enhanced drought tolerance by increasing antioxidant activity and up-regulating drought-related gene expression (Xu et al., 2017). Similarly, under salt stress, tomato plants inoculated with *P. indica* showed a significantly higher growth compared to the inoculated controls (Xu et al., 2022). *P. indica* has been shown to increase the absorption of K^+^ in tomatoes, thereby inhibiting Na^+^ accumulation, and mitigating salinity-damage of plants by improving the photosynthetic parameters (Ghorbani et al., 2018). *P. indica* could promote the growth of *Arabidopsis thaliana* under salt stress, potentially through up-regulating the expression of major Na^+^ and K^+^ ion channels (Abdelaziz et al., 2017). Although *P. indica* has a beneficial effect on a variety of herbaceous plants, there is a lack of studies on tree species.

Here we aimed to investigate the salt tolerance of paulownia mediated by *P. indica* under a gradient of concentrations, i.e., 0.00%, 0.30%, 0.50%, 0.70%. We assessed the growth and physiological performance of Paulownia seedlings and *P. indica* to analyze the growth-promoting and stress-resistant effects of *P. Indica* on Paulownia under NaCl stress as follows after establishing the symbiotic system of *P. indica* and Paulownia seedlings (1), growth of Paulownia seedlings (2), the antioxidant enzyme activity (e.g., SOD, POD, and CAT) of Paulownia seedlings, (3) osmotic adjustment substances (i.e., soluble sugar) in Paulownia seedlings, (4) photosynthetic rate of Paulownia seedling.

The causes of changes in growth and physiological and biochemical indexes of Paulownia seedlings were evaluated. We hypothesized that *P. indica* inoculation enhances salt tolerance in Paulownia by modulating antioxidant enzyme activity, osmotic adjustment, and photosynthetic efficiency.

## 2 Materials and methods

### 2.1 Plant material and growth conditions

The paulownia (*Paulownia elongata*) seeds were purchased from Shuyang Green Forest Greening Engineering (Jiangsu China). Plump seeds were selected and rinsed with deionized water. The seeds were disinfected with 75% alcohol for 30s, rinsed 3 times with deionized water, followed by 1% sodium hypochlorite for 10 min, finally rinsed with deionized water several times. The sterilized seeds were spread in petri dishes (d=90mm) covered with wet filter papers. The germinated seeds were sown into substrate (121 °C, 0.1 MPa, 2 h) peat soil (Baltic States) and perlite (Xinyang Guotong Electronic Commerce Company, Henan, China) mixture (4:1, by volume) in sterilized containers (70×67×55 mm, Jiangsu, China). Three plants are planted per container. The plants were grown in a constant environment in the greenhouse, with a temperature of 25 ± 5 °C, humidity of 50 ± 10%, and a photoperiod of 14 hours/day. The plants were fertilized weekly with Merlot nutrient solution (N:P:K=30:14:16, Shanghai Yingxi Company, Shanghai, China). Plants with similar size were selected and watered twice a week and one plant was tested per container before treatments.

### 2.2 Culture of fungi

The fungi from the Tree Stress Biology Laboratory of Alberta, Canada, were propagated in the Landscape Architecture Laboratory of Shandong Jianzhu University. Fungi were transferred to the liquid modified melin-norkrans medium (MMN) after cultured on the potato dextrose agar (PDA) medium solidified for 10 d in the dark at room temperature (25 ± 5 °C). After 7 days of shaking culture at a temperature of 26°C and a rotation speed of 150 r·min^-1^, the liquid fungi filtered and washed with deionized water for 3 times. PDA medium was prepared according to the manufacturer’s instructions (Hope Bio-Technology Co., Ltd, Shandong, China), and 25 g was dissolved in 1 L of distilled water. The medium was sterilized in autoclave for 20 min at 121°C.

### 2.3 Salinity experimental setup, fungal inoculation and treatment

A two-factor (NaCl, *P. indica*) complete randomized design (CRD) was employed for the study. The first factor, the NaCl treatment group, included a salinity solution gradient of 0.00%, 0.30%, 0.50%, and 0.70% (0.30% represents moderate salinity; 0.50–0.70%, mimics severely saline wastelands targeted for afforestation). The second factor was the treatment group of *P. indica*, including the control group (CK) without *P. indica* inoculation and the inoculation group (P) with *P. indica* inoculation. There were six plants in each treatment group, and each group was repeated 3 times. Each treatment was repeated 18 times in total among 144 individual containers (one plant/container), randomized across treatments (details in **Table 1**). All containers were clockwise exchanged every 7 days during salinity treatment, systematically changing their position relative to the light source and ventilation to balance the micro-environmental effects.

**Table 1:** CRD experiment scheme with two factors.

Concentration/(%)	Uninoculated Control Group	Inoculation Group	Total
0.00	18	18	36
0.30	18	18	36
0.50	18	18	36
0.70	18	18	36
Total	72	72	144

After two weeks of growth in liquid medium, the fungi were filtered and washed with autoclaved deionized water. The fungi were then homogenized in a blender and suspended in autoclaved water to reach the mycelial concentration of 50 (± 5) g·L^-1^. Fungal inoculum was injected into the inoculated soil of plant roots by needle tubes (Shuguang Huizhikang Biotechnology, Henan, China) with 40 ml applied per container, repeated twice in plastic containers totally.

After 7-day fungal inoculation, the inoculated and uninoculated paulownia seedlings were immersed in the corresponding NaCl solution for 1h for salinity stress treatment. All paulownia seedlings were subjected to saline immersion (30mm from the top of the pot) at 7-day intervals, and the 0.00% NaCl treatments were immersed in an equal volume of tap water under a 30-day exposure to NaCl stress. The corresponding concentration of salt water was supplemented regularly to keep the soil water content in the range of 50.00%-60.00%.

### 2.4 *P. indica* colonization inspection

At the last treatment of salt stress, four plant capillary roots (1 cm) from each treatment (40 in total) were fixed in 70% alcohol FAA (Servicebio, Hubei, China). The roots were rinsed twice with deionized water to remove the FAA from the surface. The roots were cut into about 1 cm segments and fixed on a microscope slide with ten segments randomly as a group for observation under microscope (Olympus Corporation, Tokyo, Japan) (Trouvelot et al., 1986). The colonization degree (D%) of paulownia seedlings was examined by quantifying the chlamydospore of *P. indica* in the roots as follows


(1) D(%)=I/T

where I denotes the number of infected mycorrhizal root segments; T denotes total number of examined root segments.

### 2.5 Plant mortality, biomasses, and plant growth rate

Plant mortality in each treatment was recorded following the experiment. At the end of salinity stress, the plants were harvested and weighed for fresh weight for the above- and below-ground parts. After carefully washing the root soil, the above-ground and below-ground parts were separated, and the fresh weight of the above-ground and below-ground parts of each plant was separated and weighed, respectively. Then they were put into paper bags and baked at 105 °C for 30 min, and then baked at 80 °C until constant weight, and then the dry weight of the above-ground and below-ground part was weighed respectively. Due to the inconsistent initial height, we applied the adjusted growth rate to evaluate the growth dynamics. For height growth rate of paulownia plants (R), we measured the plant height at the beginning and end of the salinity stress. The plant height was defined the distance from the soil to the highest branch as follows,


(2) R=(E−B)/B

where E is the plant height at the end of the salinity stress; B is plant height at the beginning of the salinity stress.

### 2.6 Assay of antioxidant enzymes

In each treatment group of the experiment, five strains of Paulownia were randomly selected, and the mature leaves at the same middle canopy region of each plant were sampled to determine the activity of superoxide dismutase (SOD, EC 1.15.1.1), peroxidase (POD, EC 1.11.1.7) and catalase (CAT, EC 1.11.1.6.) enzyme activities.

#### 2.6.1 Assay of SOD activity

The activity of SOD was determined by WST-8 method according to Liu et al (Liu et al., 2010), five strains of paulownia were randomly selected in each treatment, and 0.1 g of mature leaves were weighed. Following homogenization in an ice bath with 0.1 ml of phosphate buffer, the samples were analyzed using assay kits (Keming Biotechnology Co., Ltd, Jiangsu, China). According to the manufacturer’s instructions we measured the absorbance value at 450 nm with a UH5300 spectrophotometer (Hitachi, Japan) determination absorbance values to calculate respective activity values as follows,


(3) $$SOD\;\left(U/g\right)=\frac{\left(S\times V1\right)\;}{\left(W\times V2÷V3\right)}$$

S, inhibition percentage÷(1-inhibition percentage); inhibition percentage = (A0-A1)/A0; A0, denotes the reference tube suction light value; A1 is the absorbance value of the measuring tube; V1 is the total volume of the reaction system, ml; W is the sample quality, g; V2 is the sample volume added to the reaction system,ml; V3 is always the volume of added extract, ml.

#### 2.6.2 Assay of POD activity

The activity of POD enzyme in Paulownia leaves was determined by methoxyphenol method (Junfeng, 2006). The POD activity was determined by methoxyphenol method, five strains of paulownia were randomly selected in each treatment, and 0.1 g mature leaves were weighed. After ice bath homogenization with 0.1 ml phosphate buffer, the samples were analyzed with the kits (Keming Biotechnology Co., Ltd, Jiangsu, China). Then according to the manufacturer’s instructions using UH5300 spectrophotometer (Hitachi, Japan) we obtained the 470 nm absorbance values. The POD was calculated as follows


(4) $$\text{POD }\left(\text{U}/\text{g}\right)=\frac{\text{ΔA}\times \text{V}1}{\left(\text{W}\times \text{V}2÷\text{V}3\right)0.01÷\text{T}}$$

ΔA equals to A2-A1, A1 is the absorbance value at 1 min of reaction; A2 is the absorbance value at 2 min of reaction; V1 is the total volume of the reaction system, ml; W is sample quality, g; V2 is the added sample volume, ml; V3 is always added to the volume of the extract, ml; T is the reaction time, min.

#### 2.6.3 Assay of CAT activity

The activity of CAT was determined by ammonium molybdate colorimetric method (Peng et al., 2009). Five paulownia strains were randomly selected per treatment, and 0.1 g of mature leaves were sampled and weighed. After ice bath homogenization with 0.1 ml phosphate buffer, we analyzed the samples with the kits (Keming Biotechnology Co., Ltd, Jiangsu, China). Then according to the manufacturer’s instructions using UH5300 spectrophotometer (Hitachi, Japan) we determined the 405 nm absorbance values to calculate the activity (CAT) using the following formula,


(5) $$\text{CAT }\left(\text{μmol}/\text{min}/\text{g}\right)=\frac{\left(\text{ΔA}-0.0013\right)÷0.2\times \text{V}1}{\left(\text{W}\times \text{ V}2÷\text{V}3\right)÷\text{T}}\;$$

ΔA denotes A Control-A Determination;V1 is volume of the reaction system, ml; V2 is add sample volume, ml; V3 is add the volume of the extract, ml; T is reaction time, min; W is sample quality, g.

### 2.7 Soluble sugar content

The soluble sugar content in Paulownia leaves was determined by the anthrone colorimetry method (Maness, 2010). Five paulownia strains were randomly selected for treatment too. Each plant provided 0.15 g of leaf tissue; we added 1 ml of distilled water to grind into a homogenate. According to the manufacturer ‘s instructions, the absorbance at 620 nm was measured using the soluble sugar kit (Keming Biotechnology Co., Ltd, Jiangsu, China) for calculating the soluble sugar content as follows,


(6) $$\text{Soluble sugar }\left(\text{mg}/\text{g}\right)=\frac{\left[\left(\text{ΔA}+0.07\right)\;÷8.55\times \text{V}1\right]}{\left(\text{W}\times \text{V}1÷\text{V}2\right)}$$

ΔA denotes A determination; A is blank; V1 is add sample volume, ml; V2 is add the extract volume, ml; W is sample fresh weight, g.

### 2.8 Leaf net photosynthetic and transpiration rate

During the determination, nine Paulownia plants were randomly selected from each treatment for analysis. The 2–3 healthy and mature leaves of the plant from top to bottom were selected for determination. The net photosynthetic rate and transpiration rate of Paulownia leaves was measured using LI-6800 portable photosynthesis instrument (LI-COR, USA) from 9:00 am to 11:00 am and from 2:00 pm to 4:00 pm under clear weather (excluding plant photosynthesis noon break period). The environmental factors were set according to the surrounding environment, the humidity was set to 55%, the leaf chamber CO_2_ was set to 400 umol·mol^-1^, the leaf chamber temperature was set to 29°C, and the light intensity reference was 1000 umol·m^-2^·s^-1^.

### 2.9 Statistical analysis

One-way ANOVA was used to analyze the above-ground dry and fresh weight, below-ground dry and fresh weight, net photosynthetic rate, transpiration rate, SOD enzyme activity, POD enzyme activity, CAT enzyme activity and soluble sugar content of Paulownia. Levene’s test (p-value >0.05) was performed for testing the equality of variance assumption. Two-way ANOVA was used to compare treatments (including salinity stress and inoculation).The residual distribution and variances were evaluated which met the ANOVA assumptions. As described in each illustration, in a completely randomized design, experiments were performed with at least 5 independent replicates. SPSS 26.0 software was used to calculate the analysis of variance. The significance of differences between data sets was evaluated using paired student’s t-test (p-value<0.05). Statistical charts of data, i.e., bar charts, line graphs and were produced using Sigmaplot 15.0 software.

## 3 Results

### 3.1 Colonization efficiency

**Figure 1:**
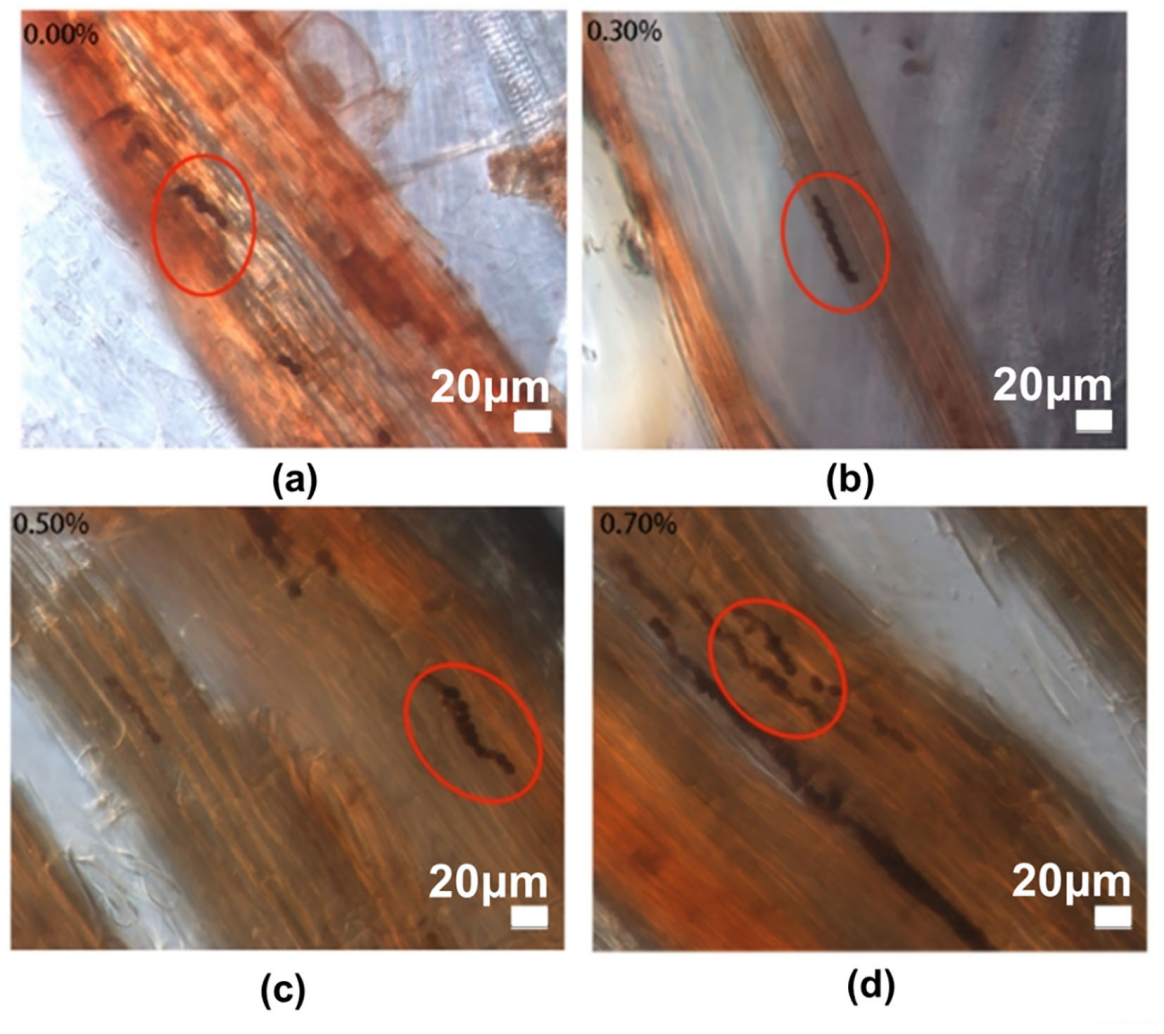
Root colonization of *P. indica* in *Paulownia elongata*. **(a)** Inoculated in 0.00% NaCl, **(b)** Inoculated in 0.30% NaCl, **(c)** Inoculated in 0.50% NaCl, and **(d)** Inoculated in 0.70% NaCl.

The colonization of *P. indica* in the roots of paulownia under different NaCl concentrations for 30 days in the inoculation group and control group (**Figure 1**). We found the chlamydospores in the roots of paulownia and confirmed the existence of *P. indica*. The colonization degree (D%) of *P. indica* ranged from 25.92% to 47.30% under 4 NaCl concentrations (**Table 2**). The D% value reached a maximum of (42.50% ± 4.79%) in plants with salinity treatment (0.70% NaCl). No chlamydospores were found in the roots of paulownia plants in the control group under all NaCl concentrations.

**Table 2:** The colonization degree (D%) of *P. indica* in paulownia root under different NaCl concentrations for 30 days.

Number	NaCl Concentration (%)	D (%) ± SE
1	0.00	30.00 ± 4.08
2	0.30	40.00 ± 4.08
3	0.50	30.00 ± 5.78
4	0.70	42.50 ± 4.79

Means (n = 40) ± standard error (SE) is shown.

### 3.2 Growth and biomass

#### 3.2.1 Mortality

The most striking effects of *P. indica* inoculation were observed in survival, growth, and stress mitigation. We found no plant mortality in the inoculated and control groups without salinity stress (0.00% NaCl). Among the three salinity stress treatment groups, as the NaCl concentration increased, the mortality increased in both the paulownia plants in the inoculated group and the control group. However, the inoculated group showed significantly lower mortality than that in the control group. Briefly, compared with the control group *P. indica*-inoculated plants showed the mortality decreased by 5.55%, 22.22% and 27.77% under 0.30%, 0.50% and 0.70% NaCl treatments, respectively (**Table 3**). The highest mortality of paulownia plants of 50.00% was observed in the uninoculated paulownia under the 0.70% NaCl treatment.

**Table 3:** Mortality (%) of paulownia plants with 5 salinity concentrations in control group (UG) and inoculated group (IG) for 30 days of salinity stress treatment (n=18).

NaCl Concentration (%)	Plant Group	Mortality (%)
0.00	IG UG	0.00 0.00
0.30	IG UG	5.56 11.11
0.50	IG UG	27.78 38.89
0.70	IG UG	38.89 50.00

#### 3.2.2 Height growth

**Figure 2:**
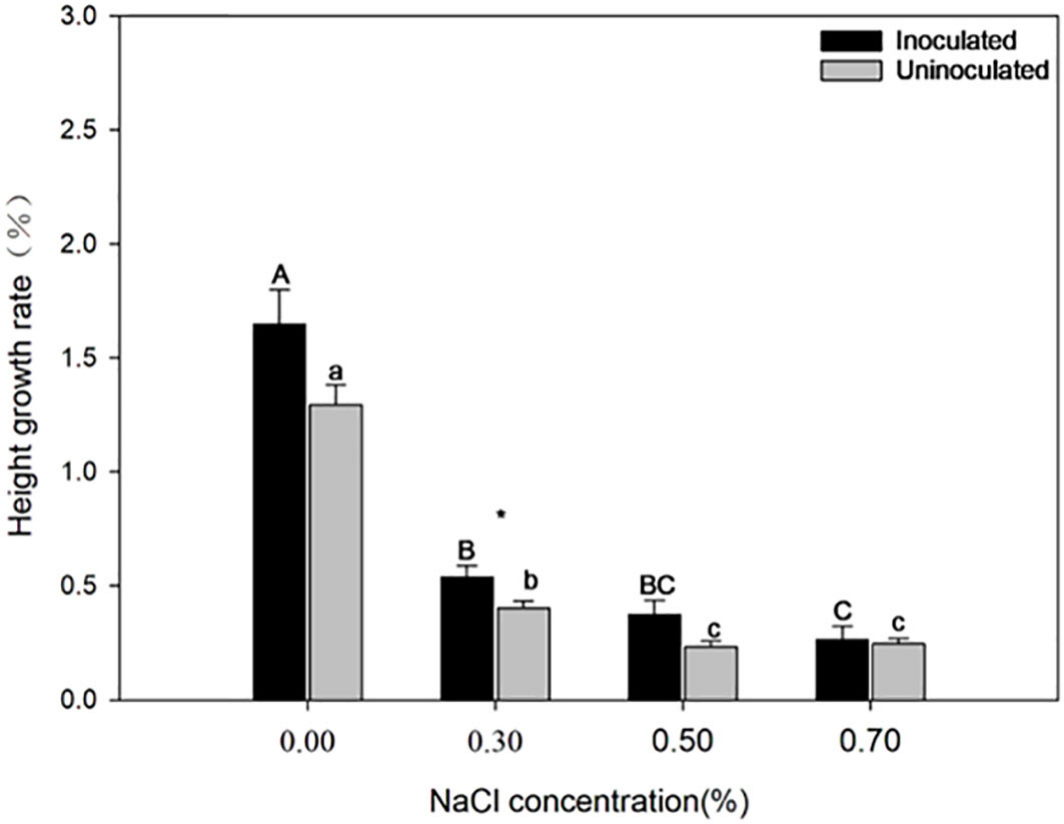
The height growth rate of paulownia plants in the inoculated group and the control group under different NaCl concentrations (0.00%, 0.30%, 0.50%, 0.70%). Note, “*”, *p*<0.05. Different majuscules indicate that there are significant differences in growth of paulownia plants in different salt concentrations for inoculated group, and different minuscules indicate that there are significant differences in growth of paulownia plants in different salt concentrations for uninoculated group.

**Figure 2** illustrates the effect of *P. indica* on the growth of paulownia plants under salinity treatment. When the NaCl concentration increased, the growth declined in both the inoculated group and the control group. However, the inoculated paulownia exhibited a higher growth rate compared to that of the control group. The inoculated group showed greater height growth under the 0.30% NaCl treatments. The plant height of paulownia decreased by 53.79%, 37.43%, 26.57% under 0.30%, 0.50% and 0.70% NaCl concentration respectively compared with that under 0.00% NaCl concentration in the inoculated group; however, the uninoculated plant height decreased by 68.88%, 81.99%, 81.00%, respectively. *P. indica*-inoculated plants showed 21.55%, 24.95%, 37.70%, and 7.41% greater height growth than controls, under the same NaCl concentration treatment (0.00%, 0.30%, 0.50%, and 0.70%).

#### 3.2.3 Biomass

##### 3.2.3.1 Above-ground fresh weight

**Figure 3:**
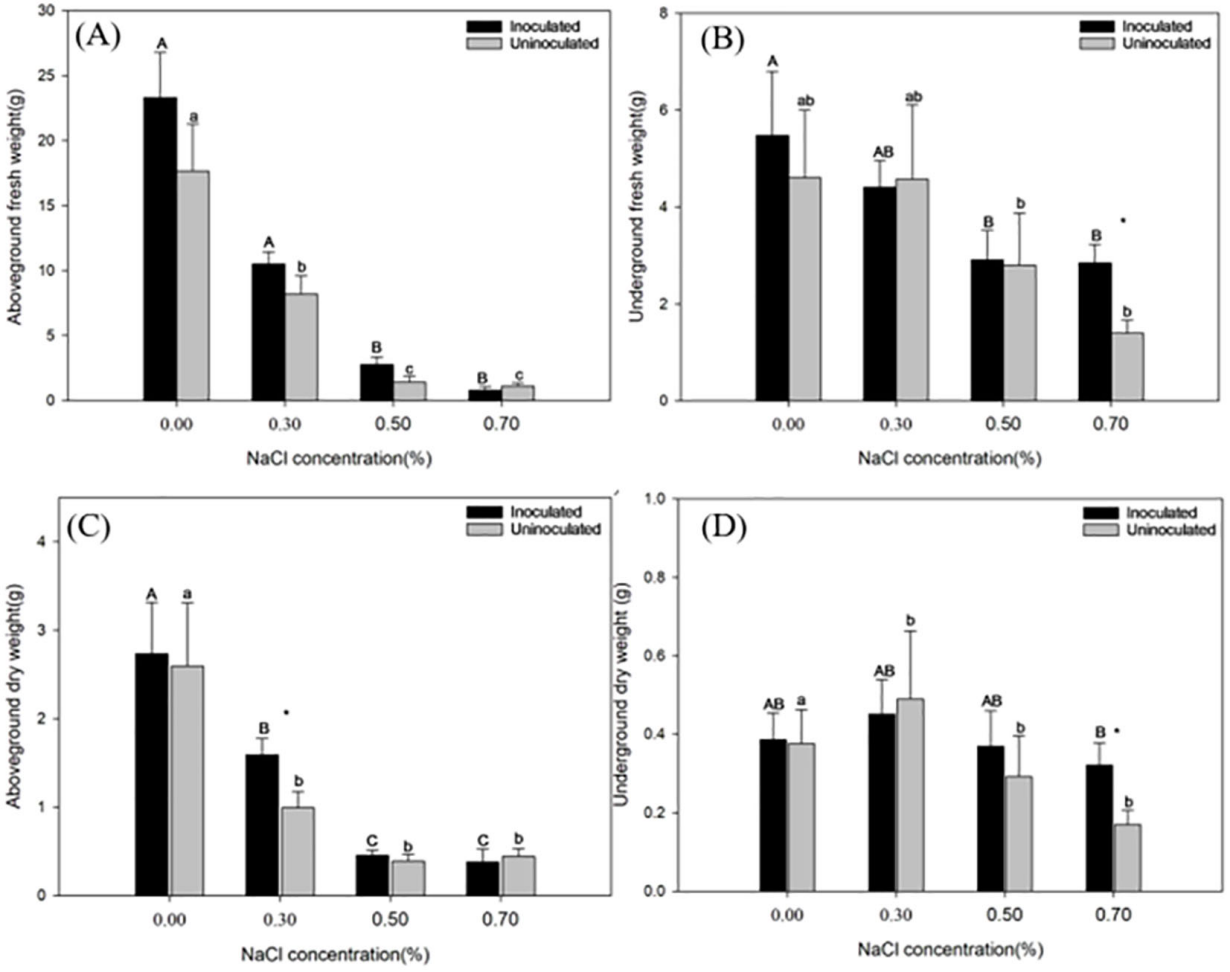
The fresh weight of paulownia plants in the above-ground **(A)** and below-ground **(B)** inoculated group and control group under different NaCl concentrations (0.00%, 0.30%, 0.50%, 0.70%). the dry weight of paulownia in the above-ground **(C)** and below-ground **(D)** for the inoculated group and control group under different NaCl concentrations (0.00%, 0.30%, 0.50%, 0.70%). Values are means (n=5) ± SE, Different majuscules indicate that there are significant differences in fresh weight of paulownia plants in different salt concentrations for inoculated group, and different minuscules indicate that there are significant differences in fresh weight of paulownia plants in different salt concentrations for control group. One-way ANOVA was performed followed by Duncan’s test (*p<*0.05); “*” indicates that there is a significant difference between the control group and the inoculated group under the same NaCl concentration stress (*p*< 0.05).

The fresh weight of plants is a key indicator of growth. As salinity stress intensifies, reduction of fresh weight is greater. **Figure 3A** reflects the changes of above-ground fresh weight of paulownia plants across rising NaCl concentration in the inoculated group and the control group. The above-ground fresh weight of paulownia plants decreased significantly with the reduction of 54.92%, 88.10% and 96.54% respectively under 0.30%, 0.50% and 0.70% NaCl concentration compared with that under 0.00% NaCl concentration in the inoculated group; in the control group, the above-ground fresh weight decreased by 49.13%, 91.17% and 92.96%, respectively, compared to the 0.00% NaCl control. Under the same NaCl concentration *P. indica*-inoculated plants showed 30.92%, 22.03%, 48.80% and -40.57% greater above-ground fresh weight than controls respectively, highlighting the mitigating effect of inoculation on salinity stress.

##### 3.2.3.2 Below-ground fresh weight

The below-ground fresh weight of paulownia plants declined with increasing NaCl concentrations (**Figure 3B**). The below-ground fresh weight of paulownia plants decreased by 17.27%, 45.39% and 46.61% respectively under 0.30%, 0.50% and 0.70% NaCl concentration compared with that under 0.00% NaCl concentration in the inoculated group. In the control group, the fresh weight decreases were more pronounced, with reductions of 6.25%, 46.64%, and 71.40% at the same NaCl levels compared with that under 0.00% NaCl concentration. Under the same NaCl concentration treatment *P. indica*-inoculated plants showed 8.49%, -3.70%, 3.89%, and 50.99% greater below-ground fresh weight than controls.

##### 3.2.3.3 Above-ground dry weight

Elevated NaCl concentrations led to a reduction in the above-ground dry weight of Paulownia plants in both inoculated and control groups. In each salinity treatment, except for 0.70% NaCl salinity concentration, the above-ground dry weight of Paulownia plants inoculated with *P. indica* was higher than that of plants uninoculated with *P. indica* (**Figure 3C**). Under the same NaCl concentration treatment *P. indica*-inoculated plants showed 8.49%, -3.70%, 3.89%, and 50.99% greater above -ground fresh dry weight than controls. Moreover, at 0.30% NaCl concentration, the above-ground dry weight of inoculated Paulownia plants was significantly higher than that of uninoculated Paulownia with *P. indica* (controls).

##### 3.2.3.4 Below-ground dry weight

The below-ground dry weight of Paulownia plants exhibited an initial increase followed by a decline as NaCl concentrations rose. Across most salinity levels, Paulownia inoculated with *P. indica* showed higher below-ground dry weight compared to non-inoculated plants, except at 0.30% NaCl. Notably, *P. indica* significantly boosted below-ground dry weight under 0.7% NaCl concentration (**Figure 3D**).

### 3.3 Effects of *P. indica* on SOD, POD and CAT activities of paulownia plants

**Figure 4:**
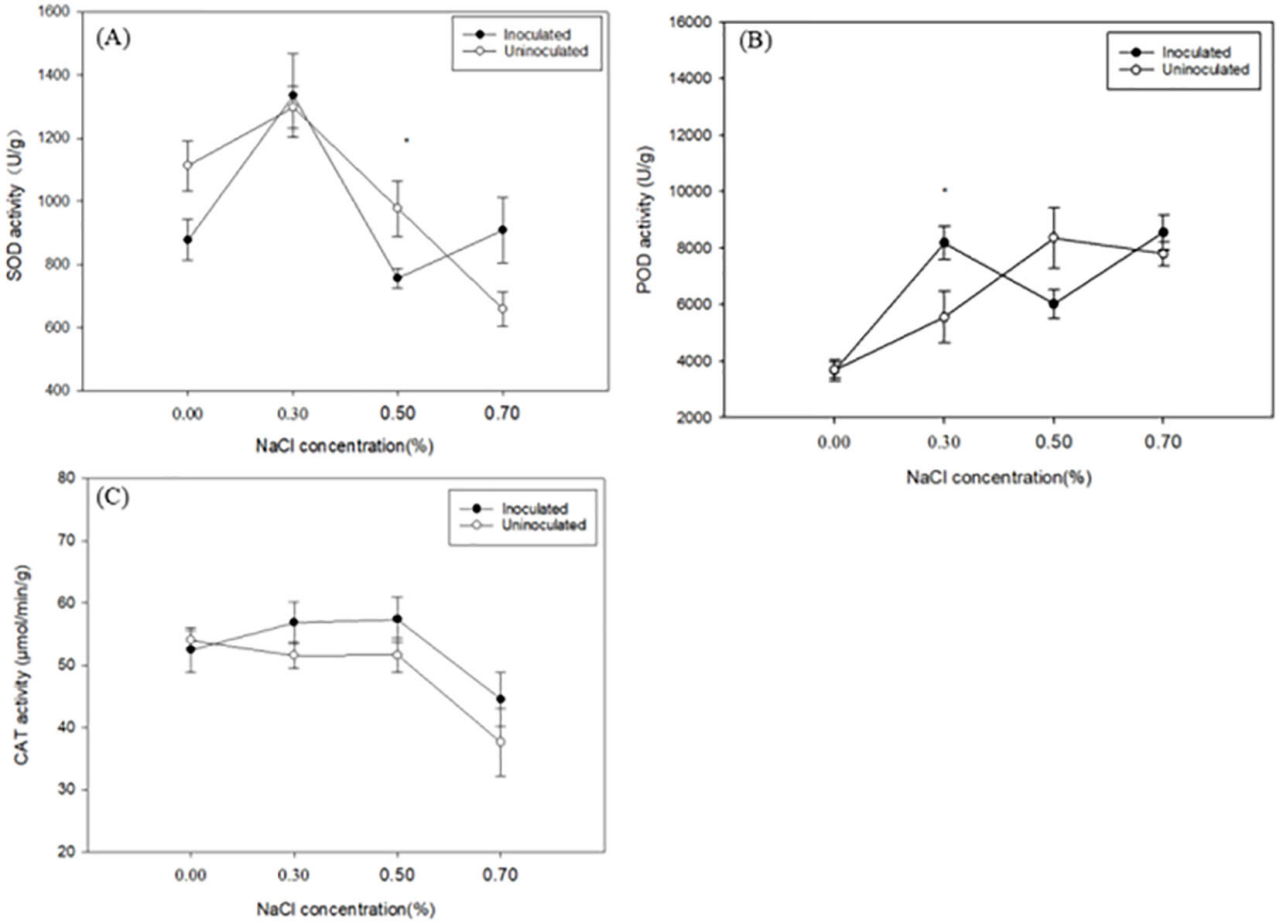
The SOD activity **(A)** POD activity **(B)** and CAT activity **(C)** of paulownia plants in the inoculated group and control group under different NaCl concentrations (0.00%, 0.30%, 0.50%, 0.70%). Values are means (n=5) ± SE. One-way ANOVA was performed followed by Duncan’s test(*p<*0.05); “*” indicates that there is a significant difference between the control group and the inoculated group under the same NaCl concentration stress (*p*< 0.05).

The activities of SOD, POD, and CAT are key indicators of a plant’s ability to detoxify reactive oxygen species (ROS). Under increasing NaCl concentrations, SOD activity in both inoculated and uninoculated plants exhibited an initial rise followed by a decline (**Figure 4A**). The SOD activity of inoculated paulownia plants under 0.30%, 0.50% and 0.70% NaCl concentration increased by 52.23%, -13.89% and 3.57% respectively, compared to the control group (0.00% NaCl). In the uninoculated plants, the SOD activity increased by 16.70%, -12.18% and -40.90% respectively, compared to the control group. Except for the plants treated with 0.50% NaCl, the increase of SOD activity in the inoculated group was higher than that in the control group. Under the same NaCl treatment *P. indica*-inoculated plants showed -26.73%, 2.84%, -29.25%, and 27.68% greater SOD activity than controls. As the NaCl concentration increased, POD activity initially rose and then declined (**Figure 4B**). The POD activity of paulownia plants peaked at 0.30% NaCl concentration in the inoculated group and was significantly higher than that in the control group (0.30% NaCl).

POD activity of the inoculated paulownia plants increased by 123.12%, 64.24% and 133.31% at 0.30%, 0.50% and 0.70% (NaCl), respectively, compared with 0.00% NaCl concentration. In the control group, POD activity increased by 50.75%, 126.87% and 111.62% under the same NaCl concentrations, respectively, compared with that under 0.00% NaCl concentration. Consistent with SOD activity, the increase of SOD activity in the inoculated group was higher than that in the control group compared with their respective 0.00% concentration of plants except for the plants treated with 0.50% NaCl. Under identical salt concentrations, POD activity in the non-inoculated group changed by -0.04%, 32.13%, -38.75%, and 8.89%. At 0.30% and 0.70% NaCl levels, SOD and POD activities in inoculated paulownia plants were higher compared to the control group, indicating enhanced antioxidant defense mechanisms in response to salt stress.

With the increase of NaCl concentration, CAT enzyme activity showed a downward trend in uninoculated paulownia, which initially increased and then decreased in uninoculated Paulownia (**Figure 4C**). CAT activity in the inoculated paulownia plants increased by 8.37%, 9.37% and -15.10% at 0.30%, 0.50% and 0.70% (NaCl), respectively, compared with 0.00% NaCl concentration. In the control group, CAT activity decreased by 4.51%, 4.45% and 30.7%, respectively compared with that under 0.00% NaCl concentration. When assessing CAT activity under identical NaCl concentrations, the uninoculated plants showed reductions of 2.13% to 0.40% at 0.30%, 0.50%, and 0.70% NaCl. Notably, except at 0.00% NaCl, CAT activity in inoculated plants was consistently higher than in uninoculated plants across the remaining salt treatments.

### 3.4 Effects of *P. indica* on soluble sugar of paulownia plants

**Figure 5:**
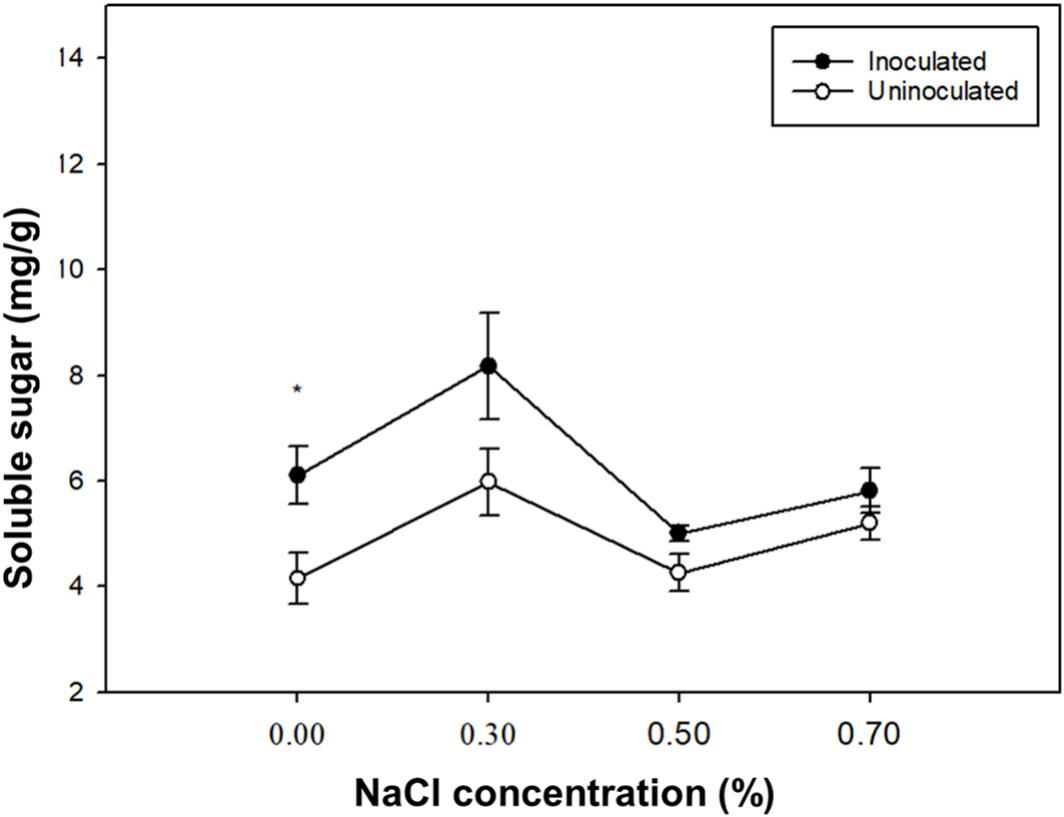
The soluble sugar content of paulownia plants in the inoculated group and control group under different NaCl concentrations (0.00%, 0.30%, 0.50%, 0.70%). Values are means (n=5) ± SE. One-way ANOVA was performed followed by Duncan’s test (*p<*0.05). * indicates that there is a significant difference between the control group and the inoculated group under the same NaCl concentration stress (*p*< 0.05).

With the increase of NaCl concentration, the soluble sugar content of paulownia plants peaked at 0.3% NaCl (**Figure 5**). At 0.00%, 0.30%, 0.50%, 0.70% NaCl concentrations, the soluble sugar content of paulownia plants inoculated group was higher than the control group. The soluble sugar content of paulownia plants under 0.30%, 0.50% and 0.70% NaCl concentration increased by 34.01%, -17.89% and -4.70% respectively compared with that under 0.00% NaCl concentration in the inoculated group, and increased by 43.89%, 2.63% and 25.20% respectively compared with that under 0.00% NaCl concentration in the control group. Under the same NaCl concentration treatment *P. indica*-inoculated plants showed -31.93%, 26.92%,14.92% and 10.58% greater soluble sugar than controls.

### 3.5 Effects of *P. indica* on leaf net photosynthetic rate and transpiration rate of paulownia plants

**Figure 6:**
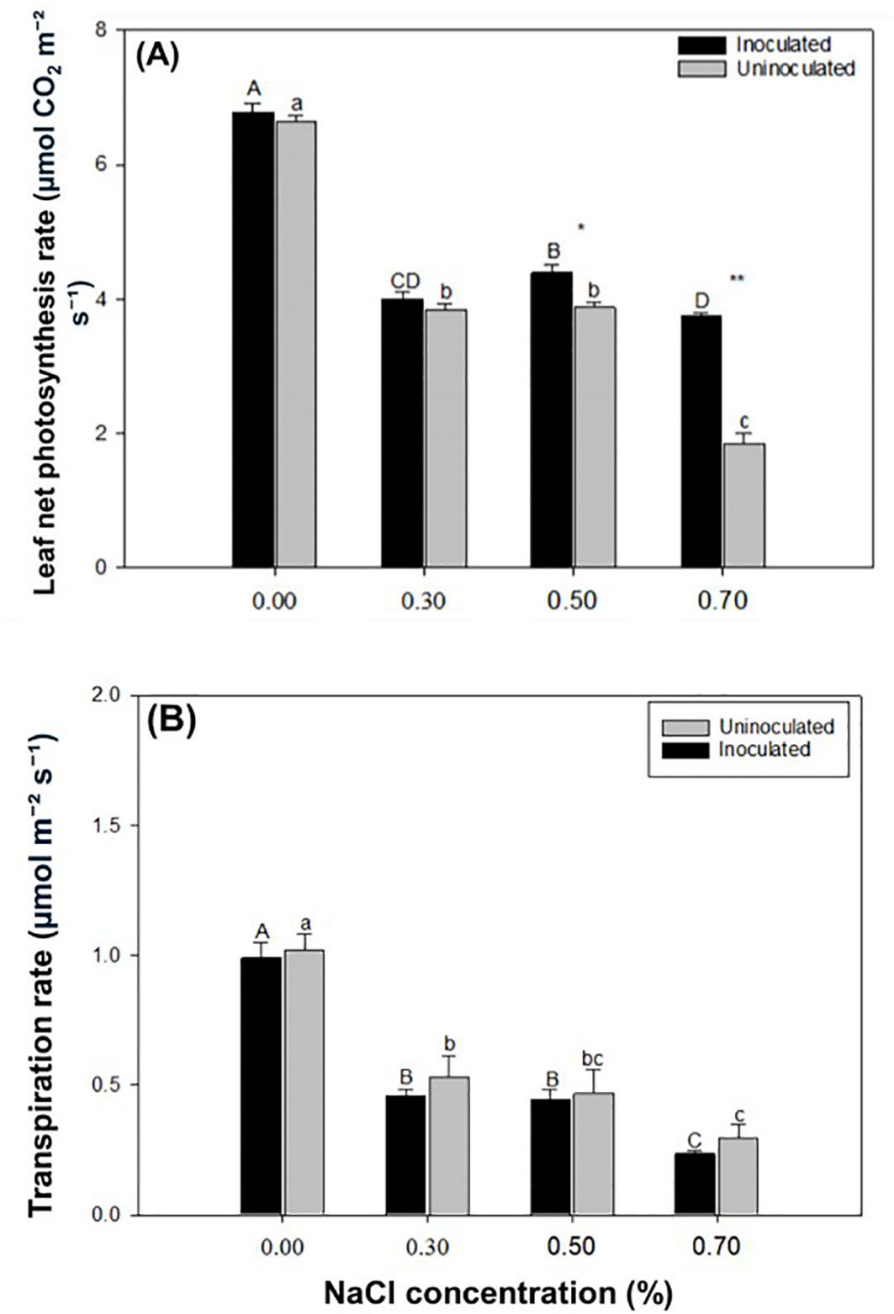
The leaf net photosynthetic rate **(A)** and transpiration rate of paulownia plants **(B)** in the inoculated group and control group under different NaCl concentrations (0.00%, 0.30%, 0.50%, 0.70%). Values are means (n=9) ± SE. Different majuscules indicate that there are signifificant differences in soluble sugar content of paulownia plants in different salt concentrations for inoculated group, and different minuscules indicate that there are signifificant differences in soluble sugar content of paulownia plants in different salt concentrations for control group. One-way ANOVA was performed followed by Duncan’s test (*p*<0.05); “*” indicates that there is a signifificant difference between the control group and the inoculated group under the same NaCl concentration stress (*p*< 0.05). ** indicates that there is a signifificant difference between the control group and the inoculated group under the same NaCl stress (*p*< 0.01).

The net photosynthetic rate of Paulownia leaves declined as NaCl concentration increased. At all NaCl concentrations, the inoculated group exhibited a higher net photosynthetic rate compared to the control group (**Figure 6**). The net photosynthetic rate of Paulownia leaves in the inoculated group was significantly higher than that in the control group at NaCl concentrations of 0.50% and 0.70%. In the inoculated group, the net photosynthetic rate decreased by 41.03%, 35.33%, and 44.88% at 0.30%, 0.50%, and 0.70% NaCl, respectively, compared to the rate at 0.00% NaCl. Similarly, the control group showed reductions of 42.15%, 41.60%, and 72.07% at the same NaCl concentrations relative to the 0.00% NaCl level. Under the same NaCl concentrations *P. indica*-inoculated plants showed 2.13%, 0.40%, 11.61% and 50.41% greater net photosynthesis rate than controls. The transpiration rate of Paulownia decreased with increasing NaCl concentration, with the inoculated group consistently showing lower rates than the control group. Under the same NaCl treatment *P. indica*-inoculated plants showed 2.95%, 14.93%, 5.36%, and 26.00% lower transpiration rate than controls.

### 3.6 Comprehensive evaluations of the response of paulownia indicators

The Paulownia indicator values were analyzed and organized, and a two-way ANOVA mean square table was constructed to evaluate the effects of *Piriformospora indica* inoculation and salt stress on Paulownia under different conditions (**Table 4**). Results revealed that the interaction between *P. indica* inoculation and salt stress had a significant effect on net photosynthetic rate and soluble sugar content, as well as a pronounced impact on plant height growth rate and SOD activity.

**Table 4:** Two-way ANOVA analysis of the response of *Piriformospora indica* to *Paulownia elongata* under NaCl salt stress.

Treatments	Salt stress x Inoculation
Plant height growth rate	3.591*
Leaf net photosynthetic rate	34.696**
above-ground dry weight	2.218
above-ground fresh weight	2.754
Below-ground fresh weight	0.872
Below-ground dry weight	0.351
CAT	0.301
POD	2.604
SOD	4.602*
Soluble sugar content	12.051**
Transpiration rate	0.070

“*” indicates significant difference at the 0.05 level and “**” indicates significant difference at the 0.01 level.

## 4 Discussion

Previous studies have shown that *P. indica* exhibits the ability to colonize a wide range of plant species. It is not only able to symbiosis with herbaceous plants such as maize (*Zea mays*) (Xu et al., 2017), barley (*Hordeum vulgare*), wheat (*Triticum aestivum*) (Ghabooli and Hosseini, 2021), rice (*Oryza sativa)* (Tsai et al., 2020), but also to symbiose with woody plants such as peach (*Prunus persica*) (Liang et al., 2023), longan (*Dimocarpus longan*) (Cheng et al., 2022), promoting the growth of host plants, and improving their ability to resist abiotic stresses such as salt and drought tolerance. However, *P. indica* had different effects on the colonization degree of different plants and plants with different initial nutritional status. The results of this experiment showed that *P. indica* could successfully colonize the roots of Paulownia and form a strong symbiotic relationship. Notably, the degree of root colonization remained relatively stable despite increasing NaCl concentrations. The colonization rates were recorded as 30.00% at 0.00% NaCl, 40.00% at 0.30% NaCl, 30.00% at 0.50% NaCl, and 42.50% at 0.70% NaCl (**Table 2**). It is well-reported that salt stress can hinder plant growth, accelerate senescence, and ultimately result in plant mortality (Jouyban, 2012). Under all salt stress conditions, the mortality rate of the inoculated group was consistently lower than that of the control group. Thus, *P. indica* inoculation improves paulownia plants to tolerate salt stress, reducing the salinity-induced damage to a certain extent.

Our results showed that paulownia plants inoculated with *P. indica* had an elevated growth rate compared to uninoculated plants at all salt concentrations. This is similar to the results for rice (Jogawat et al., 2013). Salt stress reduces plant water use and disrupts plant photosynthesis, which consequently suppresses plant growth and yield (Munns, 2002). Salt stress will also significantly reduce the biomass accumulation of plants (Pour-Aboughadareh et al., 2021). Salt stress inhibits the growth and development of plant roots, which directly limits the ability of plants to absorb water and nutrients from the soil (Nawaz et al., 2010).

We found that for the below-ground biomass, inoculated with *P. indica* significantly increased the root biomass of Paulownia under the treatment of 0.70% NaCl concentration (**Figure 3**). It indicated that *P. indica* significantly promoted the growth of the below-ground part of Paulownia under the treatment of 0.70% NaCl concentration. A similar trend was reported on gerbera (*Gerbera jamesonii*) (Chen et al., 2022) and tomato (*Solanum lycopersicum*) (Ghorbani et al., 2018). The observed growth enhancement of Paulownia following inoculation with *P. indica* is likely attributable to the ability of *P. indica* to promote plant growth by facilitating the uptake of essential mineral nutrients, particularly phosphate (Kumar et al., 2011). The unexpected biomass reduction in inoculated plants at 0.70% NaCl (**Figure 3A**) likely reflects a trade-off between survival and growth under extreme stress: Energy reallocation *P. indica* may prioritize root maintenance overshoot growth at lethal salinity (Pérez-Alonso et al., 2020), as evidenced by the 51% higher root biomass in inoculated plants (**Figure 3D**).

Plants capture light energy through leaves and convert water and carbon dioxide into glucose and oxygen. Photosynthesis process provides energy and basic substances to support various activities throughout plants’ life cycle (Pessarakli, 2024). Our results showed that the above-ground biomass of Paulownia inoculated group was higher than that of control group under all NaCl concentration treatments except 0.70% NaCl concentration treatment. After inoculation with *P. indica*, the growth of the above-ground tissues increased to a certain extent, which is consistent with previous findings in *Arabidopsis thaliana* (Abdelaziz et al., 2017). Although rhizosphere growth-promoting bacteria mainly act on roots, they can also indirectly enhance photosynthesis and improve the photosynthetic efficiency, thus fostering above-ground growth (Sun et al., 2014). Plants have developed many mechanisms to adapt to stress, such as increasing root length to promote water uptake and reducing transpiration rate (Kooyers, 2015). Our results demonstrated that the transpiration rate in the inoculated group was significantly lower than that in the control group under salt stress. This reduction in transpiration played a critical role in conserving water and improving survival chances. These findings highlight that inoculation with *P. indica* can effectively enhance water retention and plant tolerance to salt stress (Mohsenifard et al., 2017; Atia et al., 2020).

The root-to-shoot ratio of Paulownia increased under high salt stress, likely reflecting an adaptive strategy to optimize water uptake. This adjustment in biomass allocation may help to adapt to reduced water availability and decreased water use efficiency caused by salt stress (Munns, 2002). With NaCl concentration increases, the dry-fresh ratio of Paulownia plants increased, but the dry-fresh ratio of the inoculated group was always lower than that of the control group. High salt concentration will reduce the water absorption capacity of plants. This may be because under salt stress conditions, high salt concentration in the soil will reduce the water absorption capacity of plants, resulting in a decrease in water content in plants, so that the dry-fresh ratio increased (Hailu and Mehari, 2021). The inoculation of *P. indica* alleviated this phenomenon well because the epitaxial mycelium can help the plant roots to improve the water use efficiency of Paulownia by more effectively absorbing water from the soil (Augé, 2001; Asrar et al., 2012), thereby enhancing the salt tolerance of Paulownia (Boorboori and Zhang, 2022).

Under salt stress, the production of reactive oxygen species (ROS) increased, and the increase of ROS content promoted membrane lipid peroxidation, resulting in the destruction of membrane integrity, the leakage of electrolytes and small molecular organic matter, resulting in a series of metabolic disorders and damage to plants (de Azevedo Neto et al., 2006; Esfandiari et al., 2007; Lum et al., 2014). To alleviate the effect of oxidative stress caused by salt stress on plants, ROS is removed in the body through the antioxidant enzyme system such as superoxide dismutase (SOD), catalase (CAT), peroxide (POD) (Gharsallah et al., 2016). SOD is the main O^2-^free radical scavenging, and its enzymatic action leads to the formation of H_2_O_2_ and O_2_. The produced H_2_O_2_ was then removed by CAT and POD enzyme (Sachdev et al., 2021).

While *P. indica* universally upregulates antioxidant enzymes across hosts, the magnitude and stress threshold of this response vary: (Abdelaziz et al., 2019). studied the greenhouse tomato, under salt stress, and found the activities of SOD and CAT in colonized plants were significantly higher than those in non-colonized plants (Abdelaziz et al., 2019). (Arora et al., 2020). found that During salt stress, the activities of antioxidant enzymes SOD and CAT were enhanced after Artemisia annua was inoculated with *P. indica* (Arora et al., 2020). Our results showed that the activities of SOD, POD and CAT in paulownia leaves under salt stress increased first and then decreased, which is consistent with the findings in poplar (Rui et al., 2012). The activities of SOD and POD in the leaves of paulownia inoculated with *P. indica* increased rapidly and the accumulation was higher than that of paulownia uninoculated with *P. indica* at a certain concentration of salt treatment, which is similar to the research results of greenhouse tomato and *Brassica napus* (You, 2013; Abdelaziz et al., 2019). Paulownia seedlings inoculated with *P. indica* under salt stress had better H_2_O_2_ scavenging ability, reduced active oxygen content and membrane lipid peroxidation (Xu et al., 2017). With the increase of salt concentration, the activities of POD and SOD enzymes in paulownia leaves decreased, the membrane lipid peroxidation of plants increased, the activities of various protective enzymes decreased, and the growth of plants was inhibited. Low salt can induce increases of SOD and POD activity, antioxidant enzyme activity, and scavenging excessive reactive oxygen species (Liu et al., 2021). However, the activities of SOD and POD in the leaves of inoculated paulownia seedlings were still higher than those of paulownia seedlings not inoculated with *P. indica* when the NaCl concentration was 0.70%.

The activity of CAT did not change significantly. In the early stage of salt stress, plants may produce abundant superoxide anion free radicals, as the substrate of SOD, so that it is quickly activated. The hydrogen peroxide produced by SOD can become the substrate of POD, which promotes POD to respond quickly. If the hydrogen peroxide produced in the early stage of salt stress is not the main form of reactive oxygen species, then CAT cannot play a role as quickly as SOD and POD, resulting in a weak response to its activity (Joseph and Jini, 2010). Therefore, it is speculated that the enzymatic mechanism of inoculation of *P. indica* to improve the salt stress resistance in paulownia seedlings may be that SOD and POD play a key role in alleviating a series of reactive oxygen species damage which aligns with the findings in dandelion (*Taraxacum mongolicum*) (Yahui et al., 2017).

Osmotic adjustment ability plays a key role in plant salt tolerance (Munns et al., 2020). Soluble sugar is considered to be an osmotic adjustment substance that is significantly accumulated in many plants under salt stress or drought stress (Farhangi-Abriz and Torabian, 2017). Our results showed that the soluble sugar content in paulownia leaves increased first and then decreased under salt stress. The soluble sugar content of Paulownia leaves at each NaCl concentration was higher than that of the control group, indicating that *P. indica* increases the content of soluble sugar in the cell osmotic substance, reducing the cell osmotic potential, and delaying the loss of intra-cellular water (Redillas et al., 2012).

The results of two-way ANOVA also showed that under the salt-stress treatment, compared with the un-inoculated treatment, the inoculated with *P. indica* mycorrhizal fungi could significantly improve the plant height growth rate, net photosynthetic rate, and SOD activity of paulownia seedlings, thereby improving the salt-tolerance of paulownia seedlings. More in-depth studies on saline-alkaline stress are promising in the light of ROS scavenging capacity, osmotic adjustment substance content, and photosynthetic capacity, and how these regulations promote plant growth with *P. indica* inoculation for more SRWCs and bio-energy tree crops (Yahui et al., 2017). Besides, more detailed studies on the light energy utilization efficiency can benefit the understanding of the plant adaptation to the salinity conditions in terms of growth in both shoot and roots, though we provided evidence in the harvest evaluation of the productivity.

Our findings have direct implications for saline land afforestation and sustainable agroforestry: 1) Field-ready inoculant, i.e., *P. indica*’s axenic cultivability (Varma et al., 1999), enables large-scale production of low-cost inoculants; and combined with Paulownia’s rapid growth, our application could transform marginal saline soils (ECe 4–8 dS/m) into productive plantations within 3–5 years; 2) Water conservation due to reduced transpiration rates of inoculated Paulownia (**Figure 6**) can be achieved by 30–40% lower irrigation demand compared to uninoculated plants—critical for arid saline regions; 3) Regarding policy integration, promoting afforestation (e.g., China’s ‘Green Belt’ initiative) could prioritize *P. indica*-Paulownia systems in saline-alkali land reclamation programs nation-wide.

## 5 Conclusion

*P. indica* alleviates salt stress-induced mortality in paulownia plants, significantly enhancing plant height and biomass. Inoculation with *P. indica* also increases the activity of antioxidant enzymes such as POD and SOD under specific salt concentrations and boosts soluble sugar content in the leaves. These results suggest that *P. indica* mitigates oxidative damage from reactive oxygen species by enhancing antioxidant enzyme activity and protects paulownia from osmotic stress by elevating soluble sugar levels. However, further research is still necessary to verify these effects and explore the potential application of *P. indica* as a biofertilizer to improve paulownia yield under salt stress.

## Data Availability

Publicly available datasets were analyzed in this study. This data can be found here: Please contact the author for the data access.
